# The potential role of nanomedicine on COVID-19 therapeutics

**DOI:** 10.4155/tde-2020-0069

**Published:** 2020-06-29

**Authors:** Rubiana Mara Mainardes, Camila Diedrich

**Affiliations:** ^1^Pharmaceutical Nanotechnology Laboratory, Universidade Estadual do Centro-Oeste, Alameda Élio Antonio Dalla Vecchia, 838 - CEP, Guarapuava PR 85040-167, Brazil

**Keywords:** COVID-19, drug delivery, nanoparticles

Since December 2019, several cases of a different type of pneumonia were reported in Wuhan, Central China [1]. Patients symptoms included fever, dry cough, dyspnoea and gastrointestinal issues [2]. Within days, the authorities confirmed that a novel coronavirus, severe acute respiratory syndrome coronavirus (SARS-CoV-2) caused the outbreak [1]. SARS-CoV-2 virus is the agent responsible for the coronavirus disease termed COVID-19 [2].

## Main features of COVID-19

SARS-CoV-2 belongs to Coronaviruses (CoV), a ssRNA family of viruses that infect animals and humans [3]. The CoV family is organized into four genera: alpha-coronavirus, beta-coronavirus (β-CoV), gamma-coronavirus and delta-coronavirus (δ-CoV). Alpha-coronavirus and β-CoV genera are responsible for infections in mammals [4]. SARS-CoV-2 is classified as β-CoV, the same genera as SARS-CoV and middle east respiratory syndrome (MERS-CoV), the respiratory syndromes that caused outbreaks in 2002 in China and 2012 in Saudi Arabia, respectively [5]. SARS-CoV was first identified in Guangdong, China, in 2002 and infected 8098 individuals in 26 countries worldwide, leading to a mortality rate of 9.0% [2]. MERS-CoV first appeared in Saudi Arabia in 2012 and circulated along the Arabian Peninsula, resulting in 2494 cases, of which 858 were fatal, reaching a mortality rate higher than 35.0% [1]. Although SARS-CoV-2 has been reported to have over 80.0% similarity with the genome of SARS and 50.0% similarity with MERS [6], it presents higher transmission and infection rates, but a low fatality percentage, according to published reports [7]. On the other hand, the reproductive number (R_0_) of SARS-CoV-2 is estimated to be 2.2, while SARS-CoV and MERS-CoV have an R_0_ of 1.8 and <1, respectively [8]. In addition, SARS-CoV and MERS-CoV spread in hospital environment, whereas SARS-CoV-2 is transmitted in the community, due to the less severe symptoms [8]. The high R_0_ added to the large number of asymptomatic and subclinical cases, as well as lead to the great pandemic potential of COVID-19.

To infect a system, a virus binds to a receptor in a host cell, merging with the cell membrane [5]. SARS-CoV-2 and SARS-CoV have the same human cell receptor, the angiotensin-converting enzyme 2, known as ACE2, while MERS-CoV enters into the cell through the dipeptidyl peptidase 4 (DPP4) receptor [2,8]. The main difference between MERS-CoV and SARS-CoVs receptors is that the DPP4 receptor for MERS-CoV is highly expressed in the kidney, resulting in severe kidney injury [8]. The ACE2 receptor is used for SARS-CoV and SARS-CoV-2 to access the lung epithelial cell, where the virus replicates rapidly, leading to tissue injury [4]. Recent findings indicate that SARS-CoV-2 binds to ACE2 at a rate tenfold higher than SARS-CoV [7], which explains its faster human-to-human spreading [1,4]. Once the lung epithelium is damaged, the immune response soon brings about pro-inflammatory cytokines, causing acute respiratory distress syndrome and multiple organ failure [4,8]. This immune response to SASR-CoV-2 is announced by laboratory indicators, such as lymphopenia in 81.0% of patients, followed by a decrease in platelet count and albumin levels, as well as increased aminotransferases, lactic dehydrogenase, creatine kinase and C-reactive protein levels [5,7–9]. Regarding radiological characteristics, COVID-19 indicators are pulmonary lesions including bilateral ground-glass opacity in 68.5% of the cases, followed by consolidations, smooth or irregular interlobular septal and adjacent pleura thickening [7,8]. Additionally, 32.8% of patients present acute respiratory distress syndrome and 13.0% show acute cardiac injury [9].

Due to its fast spreading, the early identification of SARS-CoV-2 cases is currently the best way to prevent COVID-19 from advancing. Under this urgent circumstance, many detection methods have been developed to control the SARS-CoV-2 outbreak [10]. COVID-19 virus detection is performed using samples such as swabs, nasal swabs, nasopharynx or trachea extracts, sputum or lung tissue, blood and feces from suspected patients [3]. The most applied method of laboratory diagnosis is the nucleic acid detection through reverse transcription-quantitative polymerase chain reaction [4]. Meanwhile, the specific high sensitivity enzymatic reporter unlocking technology, developed by Massachusetts Institute of Technology, MA, USA experts, offers rapid investigation of the virus using synthetic SARS-CoV-2 RNA fragments, reducing the diagnosis time [7]. Through SARS-CoV-2 identification, the virus is differentiated from other diseases, for instance, influenza, bacterial pneumonia, adenovirus, rhinovirus and other noninfectious illness [3]. Importantly, the reduction of recognition time seems to be essential, since the rapid spread of COVID-19 [11].

## Therapeutics of COVID-19

Although all populations are susceptible to COVID-19, studies point to its prevalence in patients with comorbidities such as hypertension, diabetes, respiratory system disorders and heart illness [10]. The median age reported for COVID-19 patients is around 51.2-year old, being male in 55.9% of cases [7,9]. Studies also indicate that the disease incubation period is around 3.0–7.0 days, taking 14.0–20.0 days from first symptoms to death in fatal cases [4]. However, in people over 70-year old, this time decreases to 11.5 days [7].

So far, no treatment for COVID-19 has been considered effective and several strategies are being tested [11]. In the beginning of the global outbreak, the WHO announced that a vaccine for SARS-CoV-2 might be at hand in 18 months, however as the structure of virus has been revealed, rapid strategies targeting the spike glycoprotein have been optimized [1] and many are under clinical trials. To date, the adopted treatment is the application of broad-spectrum antiviral drugs. Among the tested antiviral drugs used is interferon, which has shown to be effective against CoVs [10,11] and has been recommended by The National Health Commission of the People’s Republic of China in addition to lopinavir and ritonavir protease inhibitors [12]. Ribavirin, a nucleoside analogue, was used to treat SARS-CoV in Hong Kong, representing another option [4] combined with protease inhibitors [12]. The National Medical Products Administration of China approved favilavir for marketing at the beginning of the COVID-19 outbreak [10]. Another drug under test is Arbidol, which reduces the reproduction of SARS *in vitro* [7]. Remdesivir, a nucleotide analogue developed for Ebola virus, was reported as a potential treatment for COVID-19, since it demonstrated the blocking of SARS-CoV-2 replication when combined with interferon or chloroquine, as well as alone [2]. Other antiviral drugs under test are nafamostat, nitazoxanide, penciclovir, oseltamivir and baricitinib [2,12]. The number of registered clinical trials using antiviral drugs for the treatment of patients with COVID-19 is 195, up to 3 June 2020.

The antimalarial drugs chloroquine and hydroxychloroquine were considered by recent publications worldwide [13] and are included in the recommendations for the prevention and treatment of COVID-19 pneumonia in several countries. These drugs alter the endosomal and lysosomal pH, preventing viral fusion and inhibit the endocytosis mediated cell uptake of SARS-CoV-2 [14]. However, the lack of results from well-performed randomized trials make it difficult to support the use of these drugs, especially considering their well-known cardiac toxicity.

Besides antiviral drugs, other approaches have been investigated to treat COVID-19. Antiviral antibodies produced in recovered patients, for example, were isolated from their blood plasma, exhibiting positive results [2]. In addition, umbilical cord blood, rich in natural killer cells and mesenchymal stem cells, represent the body’s defense activity against SARS [4]. Regarding antibiotic therapy, a broad spectrum of antibiotics are indicated, only in case the patients develop bacterial or fungal infections during advanced stages of COVID-19 [12]. In the same way, the administration of corticosteroids must be avoided, except in cases of urgency due to adverse effects [10]. Hence, a review study revealed that more than 85.5% of patients were treated with antiviral agents, while empirical antibiotics were prescribed in 90.0% of cases [6]. With the aim of testing different mechanisms to combat SARS-CoV-2, the WHO has announced a clinical trial design to be joined by doctors from around the world [13].

## The role of nanomedicine in COVID-19

Nanomedicine impacts all fields of medicine, and has been considered an important instrument for novel diagnostics, medical imaging, nanotherapeutics, vaccines and to develop biomaterials for regenerative medicine [15]. Soft nanomaterials obtained from polymers (polymeric nanoparticles), lipids (lipid-solid nanoparticles, nanostructured lipid carriers, liposomes), surfactants (microemulsion, nanoemulsions, liquid crystals) and proteins (protein nanoparticles) have been applied in nanomedicine, especially for drug delivery. The magnitude of interactions between nanomaterials and tissues/biological molecules is the base for their use for various medical applications [16]. Drug-based nanoparticles have been developed for decades, and several are under clinical trials for cancer, neurodegenerative, inflammatory, cardiovascular and infectious diseases, although only few of them are approved for human use [13]. The improvement of biopharmaceutical, pharmacokinetic and pharmacodynamic aspects of drug loading is the main tool of soft nanomaterials. Also, nanoparticles can promote specific drug targeting (passive or active targeting) and controlled drug-release rate, thereby, affecting the efficacy and safety of the treatment. Besides soft and metal nanoparticles have been applied in nanomedicine, mainly due to their various antimicrobial activities (antibacterial, antifungal, antiparasitic and antiviral) [13].

Due to the emergence of pathogenic bacteria resistant to antimicrobials, several studies have reported the efficacy of the nanotechnology-based antimicrobial therapy. Similarly, the occurrence of new viruses and their heterogeneity has also demanded innovative therapies. This way, considering specific targeting, nanotechnology opens a new avenue for antiviral therapy. The strategy of using nanoparticles to combat SARS-CoV-2 could involve mechanisms that effect the entry of the virus into the host cell until their inactivation. The blockage of the viral surface proteins may lead to virus inactivation, so targeted nanoparticles, specific to virus expressed proteins could reduce the viral internalization [17]. Metal nanoparticles have shown the ability to block viral attachment to the cell surface, leading to the inhibition of viral internalization and thereby impairing the viral replication during viral entry. Nanoparticles composed of titanium (Ti), silver (Ag), gold (Au) and zinc (Zn) have already shown results against the HIV, influenza virus, herpes simplex virus, respiratory syncytial virus, transmissible gastroenteritis virus, monkey pox virus and zika virus [13]. The mechanism of action is based on the nanoparticles binding onto the viral envelope or its protein, impairing the interaction with the host cell. The efficacy of the treatment is related to the size, shape and the surface charge of the nanoparticles, however, safety measures must be taken regarding the concentration to avoid cytotoxicity of host cells [18]. Organic nanoparticles have been used for delivering antivirals such as zidovudine, acyclovir, dapivirine and efavirenz, with the aim to improve drug bioavailability and promote efficient drug delivery and targeted antiviral activity [19]. The main limitations of antivirals are the lack of specific targeting, resulting in cytotoxicity of the host cell, which can be addressed by organic nanoparticles. The versatility of nanoparticles makes them tunable vectors for virus targeting and specific drug delivery. Antimicrobial drugs have been tested in clinical trials for COVID-19, such as chloroquine, lopinavir, ritonavir, ribavirim and remdesivir, and have demonstrated promising results against SARS-CoV-2 [4]. Nanoencapsulation of antimicrobial drugs may contribute to the development of safer treatments for COVID-19 and other viral diseases.

Although it is well-established that nanotech-based drug-delivery systems improve existing therapeutics in medicine, its application in viral diseases is underexplored and underused, as observed in the SARS-CoV-2 pandemic. Nanostructured systems can impact diagnosis, since they can improve the detection, sensitivity and increase the signal amplification specificity in polymerase chain reaction analysis; and prophylaxis as adjuvants for vaccines, as well as therapeutics for COVID-19 through the targeting of antiviral drugs [20].

In summary, nanoparticles may play an important role at different stages of COVID-19 pathogenesis, considering their inhibition potential in the initial attachment and membrane fusion during viral entry and infected cell protein fusion. Furthermore, nanoencapsulated drugs may be more efficient in activating intracellular mechanisms to cause irreversible damage to viruses and inhibition of viral transcription, translation and replication.

## Conclusion

To date, there are no specific approved drugs for treating SARS-CoV-2, and vaccines are under clinical trials. All efforts are welcome to combat the virus, and nanotech-based approaches would bring a new perspective to conventional medicine for the inhibition of virus internalization or treatment. More studies are required to understand the interface between nanoparticles and CoV, to trace a rational design of targeted therapeutics. Certainly, a pandemic involves whole health organizations and as the pathogenesis of SARS-CoV-2 is not well understood, nanotechnology could represent a convenient strategy in addition to other approaches to provide positive outcomes for COVID-19 treatment.
